# Numerical evaluation of the use of vegetation as a shelterbelt for enhancing the wind and thermal comfort in peripheral and lateral-type skygardens in highrise buildings

**DOI:** 10.1007/s12273-022-0943-7

**Published:** 2022-11-01

**Authors:** Murtaza Mohammadi, Paige Wenbin Tien, John Kaiser Calautit

**Affiliations:** Department of Architecture and Built Environment, University of Nottingham, Nottingham, UK

**Keywords:** built environment, computational fluid dynamics (CFD), wind comfort, thermal comfort, skygarden

## Abstract

Skygardens or skycourts are a unique architectural intervention in the built environment, enhancing the social, economic, and environmental values of the building. It allows occupants to connect and experience outdoor freshness within a semi-enclosed environment. However, skygardens located on a highrise building may generate intense wind gusts, endangering the safety of occupants. Using a validated computational fluid dynamics model, this study investigates the potential of various vegetative barriers or shelterbelts in attenuating the high wind speeds encountered in such spaces and the impact on wind and thermal comfort. Three skygarden configurations were investigated with and without vegetative barriers, simplified and modelled as porous zones, and their effect was studied on the velocity and temperature profile at the occupants’ level. The results indicate that while hedges and trees can offer resistance to airflow, trees provide higher temperature reduction. However, a combination of vegetative and geometrical barriers provides the most optimal condition in the skygarden. The study has identified the importance of assessing wind attenuation characteristics of tree plantations on highrise skygarden, and the results can be used in designing intervention strategies. Moreover, vegetation can attenuate pollutants and mitigate poor air quality by surface deposition, and future studies should investigate in that direction.

**Electronic Supplementary Material (ESM):**

The Appendix is available in the online version of this article at 10.1007/s12273-022-0943-7.

## Keywords built environment;
computational fluid dynamics (CFD);
wind comfort;
thermal comfort;
skygarden

## 1 Introduction and literature review

Due to the increasing urban development and population, many highrise buildings have been built over the years (Wong 2004). New sustainable designs and approaches are being developed in these buildings to address the significant energy consumption and environmental issues (Begeç and Hamidabad 2015). These include the integration of renewables, high-performance facades, rainwater collection, and biophilic designs. Vegetative measures are often employed along open roads and urban canyons to act as barriers between traffic pollution and adjacent areas. The use of vegetation, such as green walls and roofs, hedges, trees etc., improves building sustainability and helps reduce building energy demand and $CO_2$ emissions (Aboelata 2020). In addition, it improves the local microclimate through physiological aspects (Perini and Magliocco 2014), by capturing dust (Chen et al. 2017), attenuating noise (Ferrini et al. 2020) and improving human health (WHO 2016). It also influences the outdoor aero-thermal comfort characteristics (Fabbri et al. 2020).

With increasing urbanization and numbers of highrise buildings, the integration of vegetative measures in buildings has also increased. Semi-outdoor green spaces integrated within intermediary levels of highrise buildings have recently gained popularity in dense cities such as London and Singapore. They are often referred to as skycourts or skygardens. Examples of building with skygardens include the Vertical Forest in Milan, Parkroyal in Singapore (Giacomello and Valagussa 2015; Walker 2017) and the planned L20 Sky Garden proposed in Brisbane (Brisbane Development 2018), as shown in Figure 1. Such design intervention aims to improve highrise buildings' environmental, social, and economic values

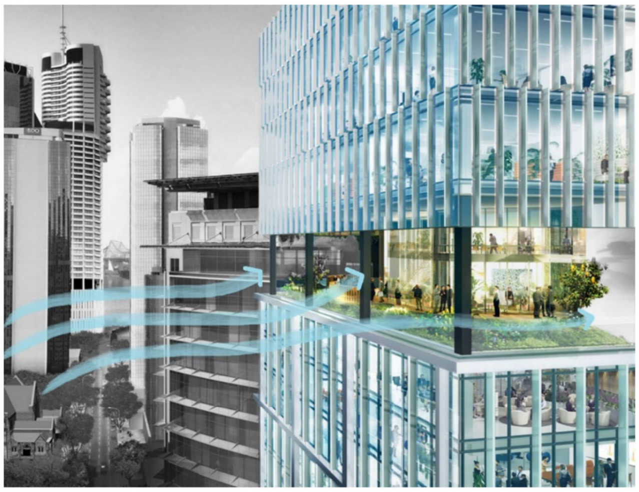

**Fig. 1** A peripheral skygarden in Brisbane, image adapted from Brisbane Development (2018)

by providing occupants with added space that allows them to connect and experience the outdoors within a semi-enclosed environment (Pomeroy 2014).

Due to the current COVID-19 pandemic, architects and engineers are rethinking the planning and design of urban and built environment spaces as it may impact the health and safety of occupants (Sharifi and Khavarian-Garmsir 2020). Vegetations, including hedges, shrubs, and trees, can act as surfaces for the deposition of aerosol particles, which are considered the primary microbe transmission mode (WHO 2009). This highlights the advantages of plants in buildings and the need to analyse their influence and impact in detail. The aerothermal conditions in skygardens, based on the impacts of different vegetation types and configurations, must be further investigated. This will help designers, architects, and engineers in selecting and arranging vegetative elements to design a favourable semi-outdoor environment for occupants residing or working in the building.

The work by Tien and Calautit (2019) investigated the influence of spatial configuration on the aerothermal performance of the skygarden. The study demonstrated that the layout and shape of the skygarden can have a significant impact on the wind distribution within the semi-enclosed environment, which can affect, both the wind and thermal comfort, of the occupants. This is further addressed in the study by Mohammadi and Calautit (2019), which integrated vegetation into the skygarden model. The results showed that the design and choice of vegetative barrier resulted in variations in wind and thermal comfort. In recent works, studies on skygardens focused on the feasibility of modifying the conditions within the space by reducing and redirecting the wind. Different types of strategies such as rails, trees and hedges were explored to enhance the wind and thermal comfort within the skygarden. To date, studies only evaluated the central-type skygarden, and further research is required to evaluate the impact of wind buffers or shelterbelts on other skygarden configurations such as peripheral and lateral-type, which are more common designs.

The addition of vegetation presented the possibility of a strategy to enhance the aerothermal performances of skygardens. There are several numerical studies of the aerothermal performance of outdoor and outdoor spaces with vegetative measures (Yang et al. 2019; Mughal et al. 2021). However, the aerothermal performance of many of the skygarden configurations, remains unknown, especially with the integration of vegetative measures. The present study aims to assess the effect of vegetation and building orientation on the aerothermal performance, using wind and thermal comfort criteria methodology across three skygarden designs; lateral, peripheral, and a hybrid design, which was introduced to address the issues observed in the previous skygarden designs. A numerical model of the skygarden within a highrise building was developed using the computational fluid dynamics (CFD) tool. Validation of the building and vegetation models was carried out using experimental data from previous works.

Two types of vegetative measures were incorporated into each of the skygarden designs – an ovoid tree and a cuboidal hedge. It should be noted that we did not attempt to explicitly model the vegetation in this study. The vegetation was simplified and simulated as a porous medium in the CFD model for the flow of air, while the cooling induced by evapotranspiration from leaves was simulated by assigning a source term to the vegetation zone. The focus was mainly on evaluating the combined impact of the skygarden design and vegetation arrangement on the wind flow speed and temperature. The study also evaluated the impact of wind direction on the aero-thermal conditions in the skygarden at the mid-height level. The wind and thermal comfort within the skygarden were evaluated using the Lawson criteria and ASHRAE-55 comfort analysis method, which used the predicted airflow velocity and temperature as input.

## 2 Methodology

The study consists of three parts, vis-a-vis, development and validation of a computational model (Section 2), generation of skygarden design alternatives (Section 3) and post-simulation analysis (Section 4). The following section describes the theory of the numerical model and the comfort model used in the analysis. We adopted the Commonwealth Advisory Aeronautical Research Council (CAARC) tall building, as it is widely researched and commonly adopted to calibrate numerical simulations of tall building designs (Braun and Awruch 2009).

## 2.1 CFD modelling approach

CFD simulations were performed using the ANSYS® FLUENT 18.1 software. The software package offers various tools for modelling, simulation and analysis of fluid flow and is widely used for commercial and academic purposes. Simulations were performed using the steady-state Reynolds averaged Navier-Stokes (RANS) realizable $k$-$\varepsilon$ model. The model has been shown to perform better than other RANS models for the specific configuration and offers better prediction in the wake of a porous medium, which has been implemented in this work and is also widely used in many urban air flow studies (Santiago et al. 2007; Gromke et al. 2015; Lee et al. 2015; Liu et al. 2015; Nazarian and Kleissl 2015; Peng et al. 2015; Toparlar et al. 2015; Yang et al. 2015; Meng et al. 2018). The governing equations are fully available in the FLUENT theory guide (ANSYS 2022), and hence, not included here. The Boussinesq approximation method was employed to account for the buoyancy caused by the temperature difference in an incompressible fluid. The discretized equations were solved by the SIMPLE algorithm, and the second-order upwind scheme was selected for discretization. The simulation was run on a workstation with a 32-core dual Xeon CPU and 32GB memory.

The test case consists of the CAARC standard tall building design, with full-scale dimensions of 45.7 m × 30.8 m × 184 m, representing the width, depth and height, respectively. Figure 2 shows the building and the computational domain. The extent of the domain was based on the guidelines suggested by Tominaga et al. (2008a, 2008b) for urban wind flow simulations. Using the height of the building, $H$, as a reference, the distances between the model and the inlet, the side, and the top surfaces of the domain were $5H$. While the distance between the model and the outlet was $15H$. An inner finer zone was also defined in the vicinity of the building, which extended up to $0.5H$ beyond the building limits.

## 2.2 Boundary conditions

Wind flow was achieved by setting one side of the computational domain as a velocity inlet and the opposite wall as a pressure outlet. The side walls were set to symmetry. The ground near the building vicinity was considered as a roughness wall, with roughness height set to 0.027 m. The atmospheric boundary layer (ABL) profile was generated using the power law model, given by Eq. (1),

$$u(z) = u(z_h) \cdot \left(\frac{z}{z_h}\right)^{\alpha} \tag{1}$$

where the wind velocity is $u(z)$ at the height $z$, the reference wind speed, $u(z_h)$, is taken at the building height ($z_h$ = 184 m) and the power-law exponent $\alpha$ is 0.33 to simulate the wind profile for cities and towns, following the study by Huang et al. (2007). Figure 3 shows the ABL implemented in the study. Turbulent kinetic energy and the dissipation rate were calculated using Eqs. (2) and (3), respectively.

$$k = \frac{3}{2} \cdot \left(u_{avg} I\right)^2 \tag{2}$$

$$\varepsilon = C_{\mu}^{3/4} \cdot \left(\frac{k^{3/2}}{l}\right) \tag{3}$$

where $u_{avg}$ is the mean velocity at the inlet, $I$ is the turbulence intensity, $C_{\mu}$ is an empirical constant, and $l$ is the characteristic length. The turbulent kinetic energy ($k$) and dissipation rate ($\varepsilon$) at the building height were calculated to be 0.8 m²/s²

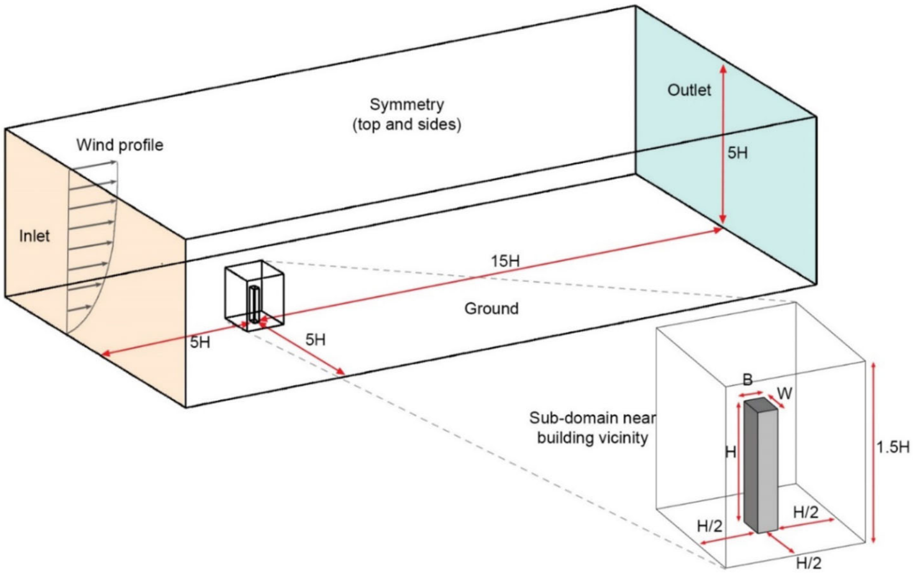

**Fig. 2** Computational domain and the inner finer zone (diagram not to scale) ($H$ is the height of the building)

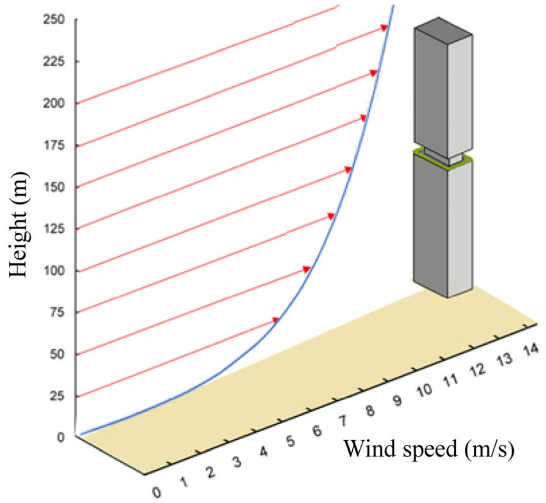

**Fig. 3** The vertical ABL profile set at the inlet of the computational domain

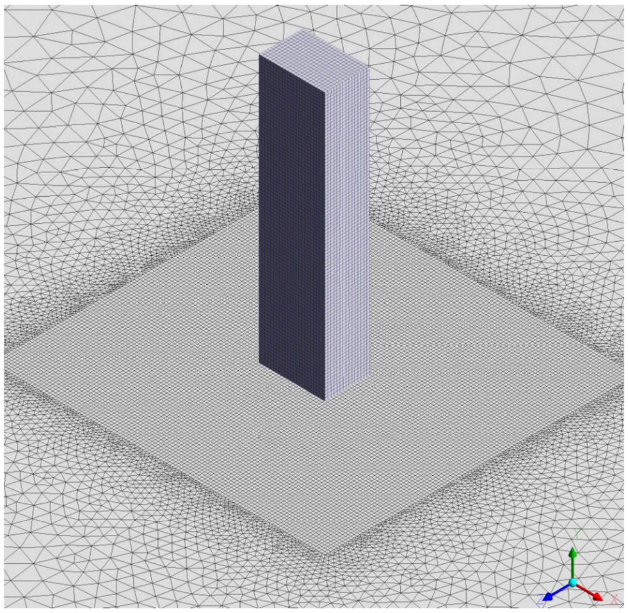

**Fig. 4** Computational mesh on the surface of the CAARC validation building and 1 $m^2/s^3$, respectively. The pressure outlet was set to 0 Pa. Although an ABL wind profile was set for the inlet velocity, we did not consider thermal stratification in this study for simplicity. The computational domain's top and side walls were set as symmetry. Gravity was set to −9.8 $m/s^2$, and the Boussinesq's approximation was turned on to account for buoyancy. The set boundary conditions of the vegetation model are covered in the next section. The walls of the building, and the skygarden, were set to no-slip wall with 0 heat flux. This was done to avoid the local thermal influence of the building as the temperature within the skygarden is affected by surface heat loss.

## 2.3 Computational mesh design

A structured mesh comprising hexahedral elements was generated in the region close to the building. While an unstructured mesh was generated in the surrounding domain. The FLUENT tool provided flexibility in mesh generation and the capability to deal with structured and unstructured mesh in its solver. As shown in Figure 2, a sub-domain was created around the building model to control the mesh settings in this region. The smallest element size is $\delta x = \delta y = \delta z = 0.0112H$, ensuring a minimum of 15 cells across its shortest edge, satisfying the sizing criteria recommended by COST action C14 (Franke 2006). The computational mesh is comprised of 6 million elements and is shown in Figure 4.

## 2.4 Validation and sensitivity analysis of the high rise building

The validation model consisted of the standard CAARC tall building (without the skygarden) where the pressure coefficient was extracted at 2/3rd of the building height, i.e., at a height of 123 m and compared with experimental data. Pressure coefficient ($C_p$) was calculated using the equation $C_p = (p - p_{ref})/(0.5\rho_{ref} v_{ref}^2)$, where $p$ is the static pressure at the point while $p_{ref}$, $\rho_{ref}$ and $v_{ref}$ are the reference pressure, density and velocity taken at the inlet. Wind tunnel measurements conducted at City University, England (CU), Bristol University, England (BU), Monash University, Australia (MU), National Physical Laboratory, England (NPL) and Tongji University, China (TJ), were used for validation and are available in the work by Meng et al. (2018). Figure 5 presents the comparison of the pressure coefficient values. The $x$-axis shows the normalized length of the analysis line, normalized with respect to the building depth ($d$ = 30.8 m), and the pressure coefficient is plotted in the $y$-direction.

The numerical simulation closely agrees with the experimental observation, especially along the windward edge, from $x$ = 0 to $x$ = 1.5. Towards the side and the rear faces, the simulation overpredicts the pressure coefficient values compared to CU, BU, MU and TJ measurements. However, the trend is fairly accurate in comparison to NPL. Deviations are the highest near $x$ = 2.5, representing the leeward corner of the building. The numerical model is unable to accurately predict the flow characteristics around the sharp corners; however, the performance is better than the standard $k$-$\varepsilon$ model, as was indicated by Tominaga et al. (2008b). The deviations in the simulated results were considered within the acceptable limit for this work, following the suggestion by Melbourne (1980). In addition, the main aim of the paper is to investigate the suitability of trees as wind buffers, the choice of turbulence offered a reasonable

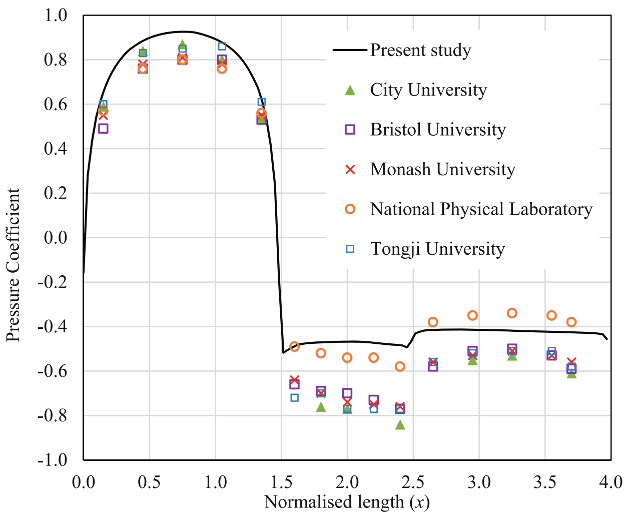

(a)

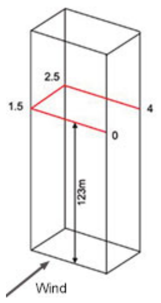

(b)

**Fig. 5** Validation of the numerical model: (a) comparison of CFD results with other experimental works, (b) location of the analysis line compromise between accuracy and time. The mesh was selected after conducting a grid verification test (see Appendix A, which is available in the Electronic Supplementary Material (ESM) of the online version of this paper).

## 2.5 Wind comfort criteria

Comfort in terms of occupancy satisfaction can be analysed in relation to both wind speeds and thermal conditions. By presenting the CFD results in terms of average wind speed, existing wind comfort criteria such as the Lawson Criteria, Davenport Criteria and the NEN 8100 Criteria could be used to assess the expected wind climate during the design stages. All three criteria provide a range of occupancy activities to evaluate the potential threshold values of wind speeds that define the skygarden regions' comfort criteria. In the present study, the Lawson criteria based on the London Docklands Development Corporation (LDDC) method (SimScale 2019) were used to provide such analysis in terms of the wind comfort across all skygarden regions of the building. Figure 6 presents the corresponding scale used to define the wind comfort criteria used in this study. Steady state CFD simulations have been performed in this work, and the wind speed at any point represents the average wind speed value. The study does not involve actual measured wind speed values from the field.

The results of the wind comfort evaluation are certainly dependent on the level or height of the skygarden in the building and hence must be taken into account when evaluating the results presented here. The skygardens evaluated here are located at mid-height of the building; clearly, the wind comfort will be different if the sky garden is located on the lower or upper floor levels. The methods and findings presented in this study may be used to aid the design of skygardens in different locations. It must also be kept in mind that the implemented boundary conditions represent a typical windy day, such that the performance of the vegetation can be evaluated on the higher end.

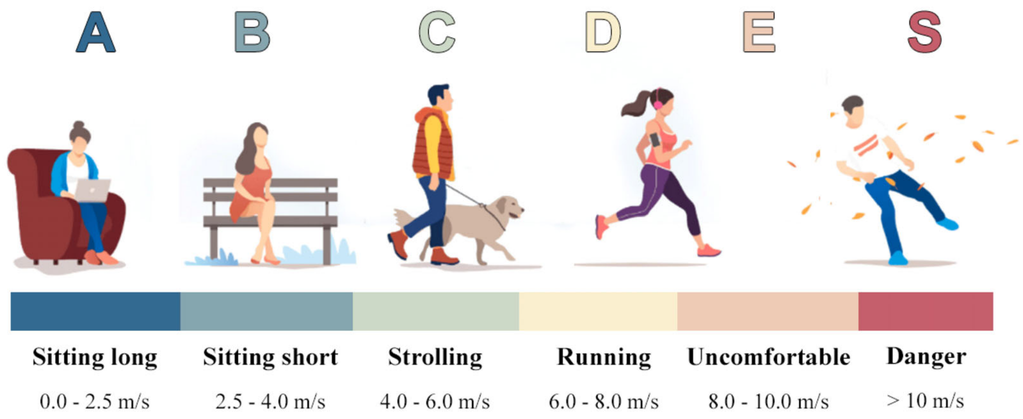

**Fig. 6** Modified Lawson LDDC wind comfort criteria (SimScale 2019)

### 2.6 Thermal comfort criteria

In addition to wind comfort, thermal comfort is equally important. The occupants' thermal sensation is quite crucial which can modify occupants' behaviour and their activities within the skygarden area. The CBE thermal comfort tool (CBE 2020) which complies with the ASHRAE-55 (ASHRAE 2020) was used to perform the thermal comfort analysis for all simulation cases. In order to evaluate the cooling potential of the vegetative measures, the building was assumed to be located in Hong Kong, which has many examples of skygardens within highrise buildings and has a warm-humid climate. An average air temperature of 30 °C and cloud cover of 68% was assumed, representing a typical hot day in Hong Kong. Occupants within the skygarden region were assumed to wear typical summer clothes, represented by a clothing value of 0.5 clo. Furthermore, a metabolic rate of 1 met was assumed, typical of sedentary activities such as sitting. Given that skygardens are semi-outdoor spaces frequently used as a social space within tall buildings, the thermal comfort across these spaces is analysed based on the standard effective temperature (SET). According to the ANSI/ASHRAE Standard 55-2010 (ASHRAE 2010), SET is the dry-bulb air temperature of a hypothetical environment at 50% relative humidity for occupants wearing clothing that would be standard for the given activity in the actual environment. It assumes a standard environment in which both air and surface temperatures are the same, and the air velocity is below 0.1 m/s. This is a comfort index that is calculated based on both the basic personal and environmental factors of thermal comfort. Based on the study by Gamero-Salinas et al. (2021), which focused on evaluating the thermal comfort of semi-outdoor environments across highrise buildings, it suggests SET as a suitable parameter to evaluate the conditions in the proposed skygarden cases. It should be noted that the thermal comfort evaluation in this study is rather simplified and mainly focused on the predicted airflow velocity and temperature, while other environmental and personal factors were fixed. This was due to several modelling and computational resource limitations which will be discussed in detail in Section 3.

## 3 Skygarden designs and vegetation model

### 3.1 Vegetative barrier model

The vegetative barriers were simulated as porous objects which are permeable to the wind flow. Several studies have adopted this approach to understand the behaviour of trees in urban studies, such as for pollution dispersion (Buccolieri et al. 2009; Li et al. 2016), wind shelter (Rosenfeld et al. 2010; Bitog et al. 2012; Kang et al. 2017; Kang et al. 2020), temperature amelioration (Gromke et al. 2015), etc. Several factors can affect the aerodynamic properties and cooling ability of the vegetation, such as the species type, shape, leaf thickness, leaf area index, etc., and can be complex to implement numerically. Nevertheless, for urban applications, researchers have adopted average values of these parameters to mimic the effect of vegetation. They may not be accurate for studying plant behaviour, but provide a fair representation for other applications, as was shown by Gromke and Ruck (2012) and Salim et al. (2011a). The focus of this work was to investigate the wind attenuation effect of vegetation and its thermal capabilities in semi-indoor spaces, and for such investigations, the vegetation was modelled as a porous body with source terms to account for the aerodynamic properties. It was assumed that the vegetation was healthy, watered adequately and not under any environmental stress. This method provides acceptable results for comparison between various factors such as arrangement, the distance between the vegetation, height, etc., on the wind flow. The turbulence field is approximated in this approach.

In FLUENT, the vegetation was modelled as porous media, and a cooling potential of 350 W/m$^3$ per leaf area density (LAD) was set as a source term in the energy equation following the work by Rahman et al. (2011). Following the study of Bitog et al. (2011), the vegetation geometry was modelled as shown in Figure 7 with a LAD of 2.3 m$^2$/m$^3$. The Ergun equation was used to establish the viscous resistance factor ($1/\alpha$) and the inertial resistance factor ($C_2$). FLUENT theory guide (ANSYS 2017) provides these formulae as:

$$\alpha = \frac{d^2}{150}\frac{\text{Ø}^3}{(1-\text{Ø})^2} \tag{4}$$

$$C_2 = \frac{3.5}{d}\frac{(1-\text{Ø})}{\text{Ø}^3} \tag{5}$$

where particle diameter, $d$ is set to 0.02 m based on the work by Sonnenwald et al. (2016), and void fraction Ø is set to 0.96 after a range of values between 0.90 and 0.99 were tested for best fit. The particle diameter can be loosely

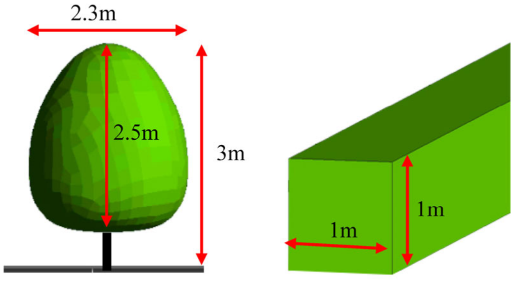

**Fig. 7** Computational domain and the vegetation size: (a) trees, (b) hedge translated as the average width of the leaves in the vegetation.

## 3.2 Vegetation model validation

A separate study was conducted to validate the vegetative barriers, based on the work by Manickathan et al. (2018). For this purpose, a 2D domain extending 35 m in length and 11.5 m in height, was created wherein the vegetation was represented as a 1 $m^2$ square placed 0.5 m above the ground and 8.5 m from the inlet. The boundary conditions were replicated as in the original work, where the inlet velocity is described by the log law profile given by Eq. (6)

$$U = \frac{u_*}{\kappa}\ln\left(\frac{z+z_o}{z_o}\right) \tag{6}$$

where $\kappa$ is the von Karman number set to 0.41, $z_o$ is the aerodynamic roughness height set to 0.0217 m, $u_*$ is the friction velocity set to 0.078. Figure 8 gives the summary of the domain settings. For the validation study, the inlet air temperature is set to 30 °C.

Figure 9 shows the velocity and temperature distribution in the vicinity of the vegetation. The resistance offered by the vegetative body can be visualised by the reduced velocity in its wake. The porous body behaves like a momentum sink, slowing airflow. Simultaneously, the temperature is also

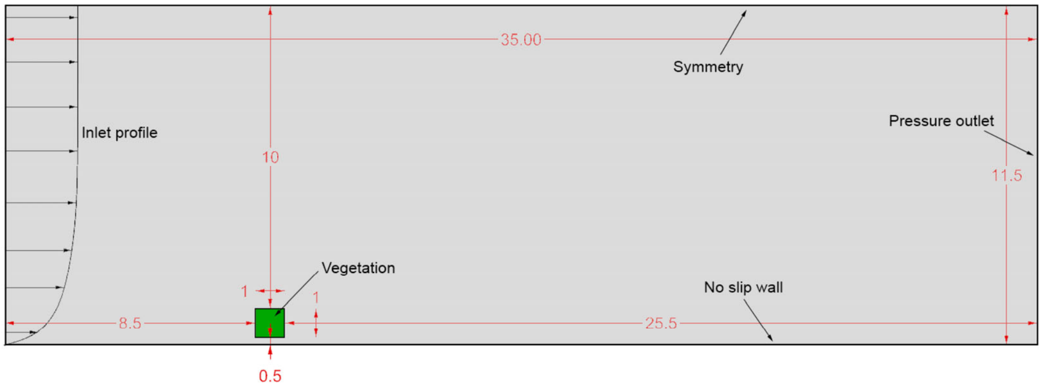

**Fig. 8** Domain description for the validation study of the vegetation

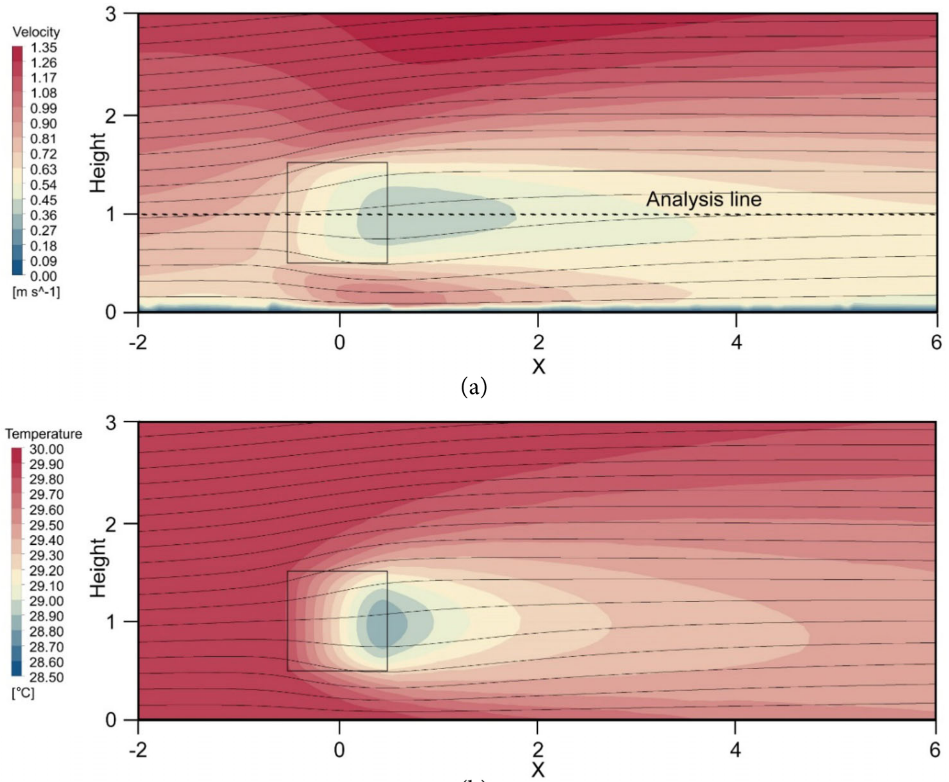

**Fig. 9** (a) Velocity and (b) temperature distribution in the vicinity of the vegetation

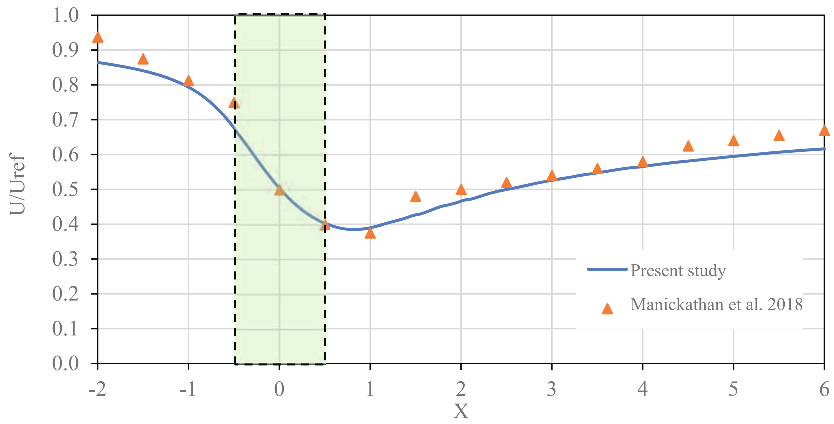

**Fig. 10** Validation of the vegetation model lowered in the wake of the vegetation as it provides a heat sink as well, leading to a drop of 1.5 °C near the leeward edge of the body.

Velocity is extracted along a line passing through the centre of the vegetation patch (marked in Figure 9(a)), and normalised with respect to the reference velocity. Results are compared in Figure 10, wherein the green patch represents the vegetation, extending from −0.5 to 0.5 on the $x$-axis. The trend closely follows the numerical model by Manickathan et al. (2018), although slight underprediction occurs in the wake of the porous zone. A deviation of about 0.05 is observed towards the far end at $x = 6$. The performance of the model is satisfactory within the ambit of modelling complexity, and given the objective of the present study to determine the attenuation effect of vegetation in skygardens, the results were deemed to be acceptable for further analysis.

It should be noted that we did not attempt to explicitly model the vegetation or tree in this study. The focus was mainly on evaluating the combined impact of the skygarden design and vegetation arrangement on the wind flow speed and temperature. The vegetation was simplified and simulated as a porous medium in the CFD model for the flow of air, similar to the studies by Salim et al. (2011b) and Jeanjean et al. (2015), etc. The cooling induced by evapotranspiration from leaves was simulated by assigning a source term to the vegetation zone. Many of the previous studies, such as Gromke et al. (2015) and Yang et al. (2019), modeled realistic spaces or case studies with different types of vegetative measures. This may have a different cooling effect as compared to the simplified arrangement of trees or hedges in this work. The present modelling technique does not consider the shading benefits of the vegetation, and the source terms do not account for turbulence or humidity.

We do acknowledge that a more realistic model of the vegetation or tree is required to fully evaluate its behaviour and effectiveness in enhancing thermal comfort, these will be carried out in future works. It should be noted that these studies would require integrating the species mass transfer model and radiation model into the already complex and computationally intensive model of the skygarden building. Finally, we have also attempted to model the shading effect of trees in the FLUENT CFD tool, but there is currently an issue with the porous model (for the vegetation) not participating in the solar radiation modelling. FLUENT treats the porous region as a transparent zone, and the thermal radiations do not interact. Perhaps this can also be tackled in future studies. A short review of experimental studies evaluating the impact of vegetative measures on solar shading and thermal comfort is presented in Appendix B, which highlights the difficulty in modelling this aspect using numerical codes. The Appendix is available in the Electronic Supplementary Material (ESM) of the online version of this paper. However, as mentioned previously, the present model should be adequate for evaluating the combined impact of the skygarden design and vegetation arrangement on the wind flow speed and temperature as a preliminary study.

## 3.3 Skygarden configurations

According to Pomeroy (2014), the interstitial or the peripheral, skygarden is a case where the spatial configuration of the building provides an open space along the perimeter. The lateral or the side skygarden is a modified version, wherein the open space is only along one edge of the building (Alnusairat 2018), such as 20 Fenchurch Street in London or the proposed L20 Sky Garden proposed in Brisbane (see Figure 1). Examples of peripheral skygarden include East 44th Street in Manhattan, proposed by ODA New York (http://www.oda-architecture.com/projects/east-44th-street). Figures 11 and 12 show the two skygarden configurations evaluated in the present work; peripheral and lateral (side) types, where both have equal areas in terms of outdoor green space. The skygarden was located at the mid-height of the

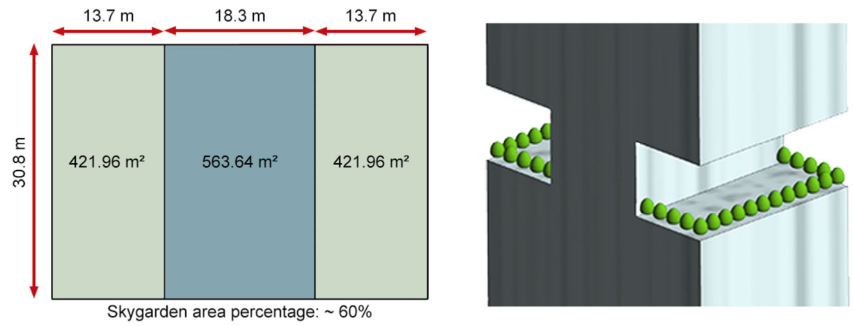

(a) (b)

**Fig. 11** Lateral skygarden design: (a) configuration and area, (b) perspective view

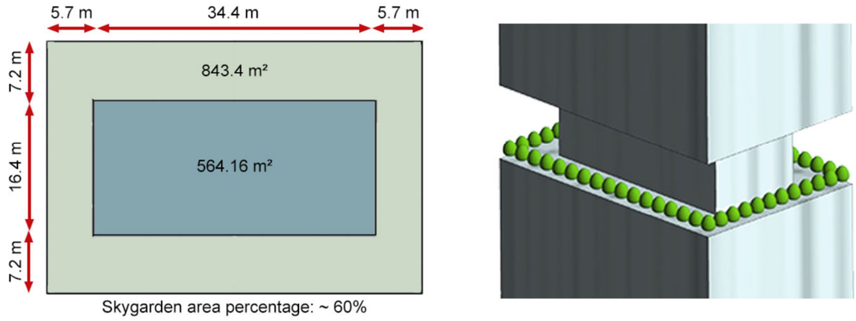

(a) (b)

**Fig. 12** Peripheral skygarden design: (a) configuration and area, (b) perspective view building, i.e., at $H = 92$ m. The height of the skygarden is 12 m, which is about 3 storeys. In order to simplify the geometry, the skygardens were modelled with only the vegetation, while other components such as structural supports, furniture, etc. were excluded.

The two skygarden designs were implemented on the building geometry, such that the area covered by the skygardens were similar in both cases, i.e., 843 m$^2$. This was approximately 60% of the building footprint. For each design, three cases were studied. One without any wind barriers or railings, representing the base case (none), one with the presence of a 1 m high hedge along the boundary and one with the addition of 2.5 m high trees. Three building orientations were simulated to investigate the conditions under different wind directions; consequently, the buildings were oriented at an angle of 0°, 45° and 90° with respect to the inlet.

## 3.4 Modified (combined) skygarden design

Post assessment, the design was slightly altered to test the impact on wind comfort and thermal characteristics. A hybrid design (Figure 13) was generated combining both skygarden types, wherein the continuity of the interstitial skygarden was broken around the corners, and the sided skygarden was replicated on all edges. Vegetative barriers and wind direction were parameterised similar to previous simulations. The area covered by the skygarden was, however,

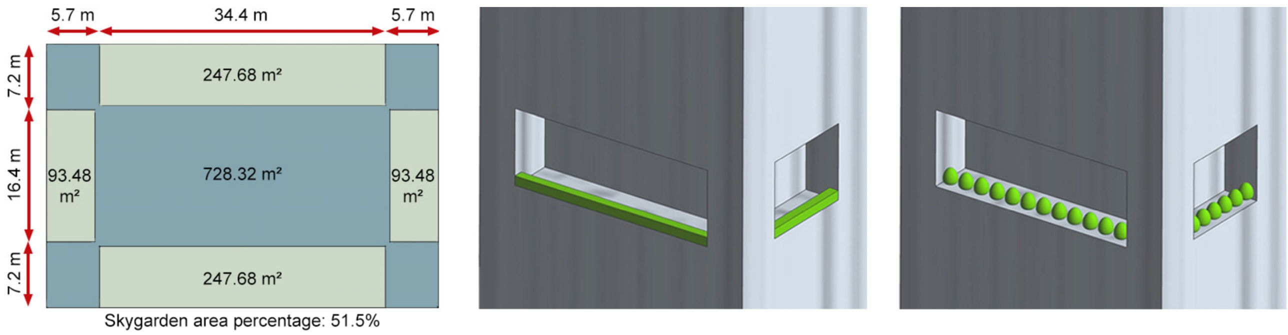

(a) (b) (c)

**Fig. 13** Modified (combined) skygarden design: (a) configuration and area, (b) building with hedges, (c) building with trees

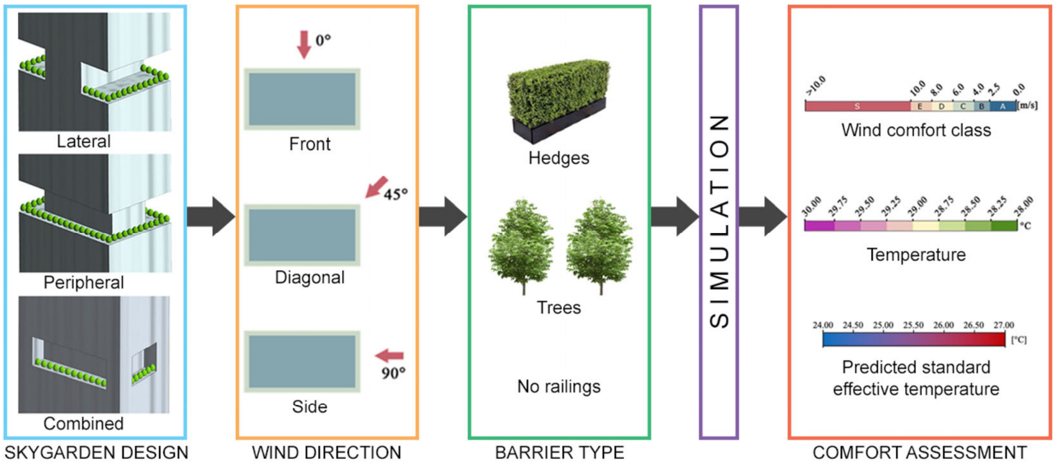

**Fig. 14** CFD analysis methodology for evaluating various designs and parameters of the skygarden reduced by 8.5% due to the presence of the corner blocks.

In summary, three skygarden configurations were generated, and each option was implemented with three types of wind attenuators. All nine cases were simulated under three wind directions. Following this, wind and thermal conditions were analysed from the 27 simulated cases. Figure 14 shows the sequence of study.

## 4 Results and discussions

The following section presents the wind and thermal comfort results across the various skygarden designs; lateral, peripheral and the combined skygarden designs. For each configuration, a total of nine simulations were carried out, representing the variations in the modes of wind barrier and wind direction employed across the skygarden region of a highrise building. These included hedges, trees and barrier-free configuration, whereas the wind was configured to flow at an angle of 0°, 45° and 90° with respect to the building. For each case, wind comfort analysis was performed at occupants' chest height (1.4 m above the floor plane) across the skygarden. Results in terms of the velocity across the different simulation cases were extracted and correlated to the wind comfort criteria as described in Figure 6. It must be kept in mind that the implemented boundary conditions represent a typical windy day, such that the performance of the vegetation can be evaluated on the higher end. In combination with wind velocities and the assumptions made, thermal comfort analysis based on the standard effective temperature (SET) was performed.

### 4.1 Lateral skygarden design

Table 1 presents the wind speed across the lateral skygarden designs with different wind angles and variations in vegetation. Generally, the wind direction played a significant role in determining the overall wind comfort in the skygarden. For all three barrier types (none, hedge and trees) when the wind was incident at 0°, near-symmetrical results were obtained on the two patches of the skygarden. While, for 45° and 90° incidence angles, large variations in wind speeds were observed across the entire region, with higher speeds on the windward side as compared to the leeward side.

The results also indicate that the generated wind speeds can cover the full comfort range from A through to S, i.e., from 0 m/s to over 10 m/s. This suggests that the lateral design with the building mass located between the two outdoor areas will force the wind to be displaced upwards and around the sides. This can be seen in the case of 0° wind direction (Table 1 – (a), (d) and (g)), where most of the skygarden region falls within the S category, with velocities over 10 m/s. Only the region close to the wall achieved lower velocities and consequently better comfort class. Furthermore, the air was then pushed downwind onto the leeward side. For all cases with the wind at an angle of 45° and 90°, it indicates a decrease in velocity with areas of reverse flow resulting in more space on the leeward side to achieve wind quality class A. Regardless of the barrier type, wind speeds are dangerously high in the skygarden when the wind direction is at 0°, and most parts of the area are covered by class S. This suggests that wind flow direction can present high variation in wind comfort for this type of skygarden design and is a determinant factor.

Since wind flow direction cannot be controlled, architects and/or designers should assess the wind and environmental conditions for the given location prior to the proposal of such lateral skygarden designs. For instance, a skygarden building built at an orientation such that the predominant wind direction is at an angle of 0°, would not be acceptable in terms of wind comfort. However, if a skygarden building were built at an orientation where the general wind condition

**Table 1** Contours of wind speed (mapped against the comfort criteria) taken at occupants' chest height for the lateral skygarden

| Lateral skygarden: wind comfort class | | | |
|---|---|---|---|
| | Vegetation type | | |
| Angle | None | Hedge | Trees |
| 0° | wind  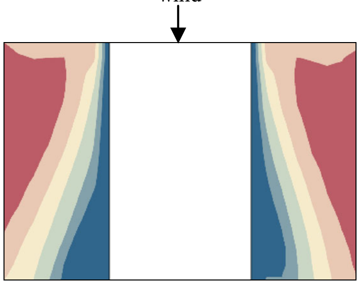 (a) | wind  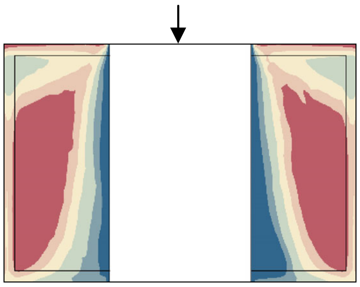 (d) | wind  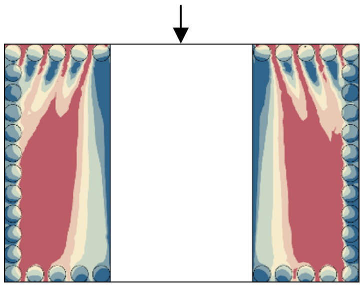 (g) |
| 45° |  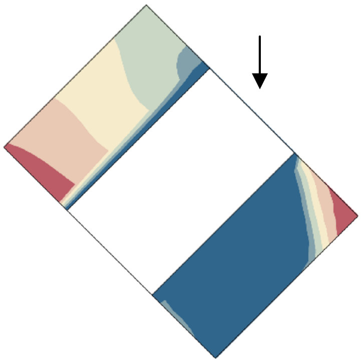 (b) |  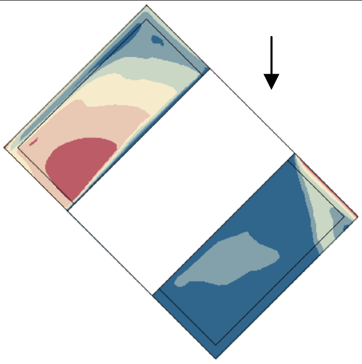 (e) |  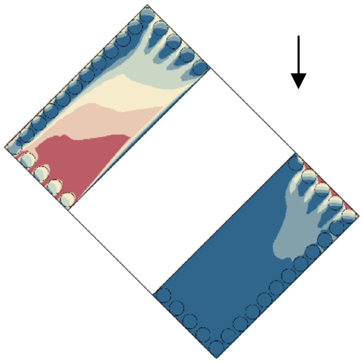 (h) |
| 90° |  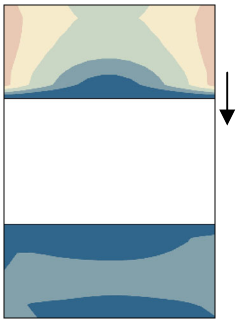 (c) |  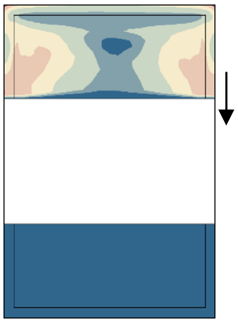 (f) |  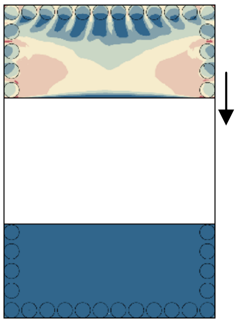 (i) |

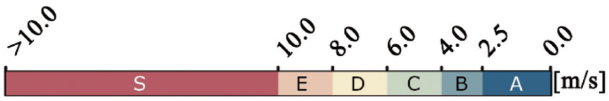

follows the results achieved for angle 45°, only a small portion of the skygarden area will not be acceptable (with wind speed greater than 10 m/s), but the placement of trees further minimised this. Following this, it indicates that lateral skygarden buildings with wind flow similar to the cases of 90° would achieve wind speeds within the acceptable range. The presence of vegetation in the skygarden region significantly reduces the wind speeds around the area nearest to it. The type of vegetation placed in the skygarden area can impact the distribution of airflow speed and direction. This is shown by the different patterns achieved by hedges and trees. Hedges considerably reduced wind speed as compared to trees. The intermittent configuration of trees resulted in lower speeds around regions immediately in its wake, in contrast to speed in the inter-tree gaps, which remained high.

From previous works analysis (Tien and Calautit 2019), it indicated that skygardens with no vegetation (known as skycourts), had minimal variation in terms of temperature. No heat sinks were present in the simulation domain to account for any discernible temperature change. Hence, for the present work, configurations with vegetative barriers alone were analysed in terms of temperature.

Table 2 presents the temperature distribution across the lateral skygarden design when hedges or trees are employed as wind attenuators. Results show that the areas where vegetation is placed show a reduction in temperature by up to 2 °C. Higher temperature reductions are observed in the vicinity of these elements. However, most of the skygarden areas did not show any significant reduction in temperatures in most cases. This indicates that there would be less variation in terms of temperature distribution for such lateral skygarden design with the given vegetation configurations. A greater volume or covered area of vegetation would be required to achieve a perceivable reduction in temperature.

**Table 2** Contours of temperature taken at occupants' chest height for the lateral skygarden

| Lateral skygarden: temperature | | |
|---|---|---|
| | Vegetation type | |
| Angle | Hedge | Trees |
| 0° |   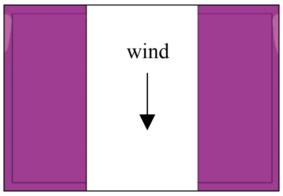 (a) |  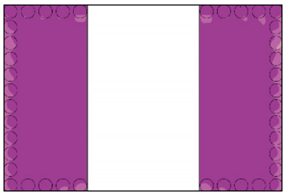 (d) |
| 45° |  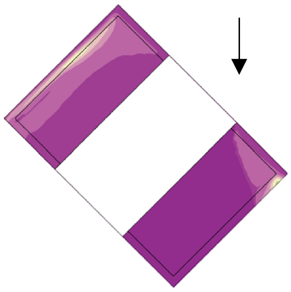 (b) |  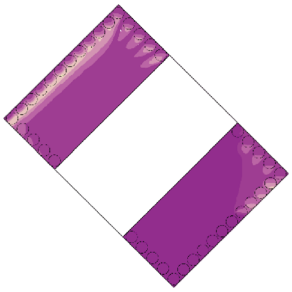 (e) |
| 90° |  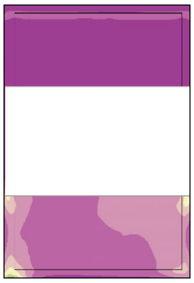 (c) |  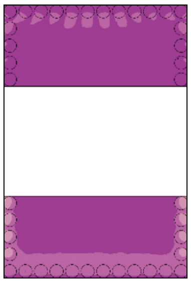 (f) |
|   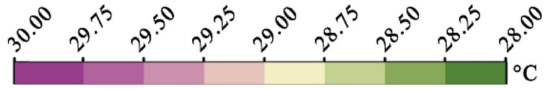 | | |

Overall, the results for the lateral skygarden indicate the greater importance of the wind angle in relation to the skygarden building rather than the area of vegetation. The wind angle at 0°, indicates that the skygarden building would become dangerous and unsuitable for occupants to be present in most areas. If, however, the wind angle was at 45° or 90°, then approximately half of the windward side, along with the whole of the leeward side, would be suitable for occupants to engage in various activities while still achieving satisfactory wind comfort.

## 4.2 Peripheral skygarden design

This section presents the analysis of the results obtained for the peripheral skygarden design. The contours of comfort class and temperature are shown in Table 3 and Table 4, respectively. For all configurations, high wind speeds are observed near the windward side of the skygarden. Further deterioration of comfort class occurs around the corner wherein the speeds are greater than 10 m/s. However, the comfort class improves towards the centre with quality class transitioning from S to A. This enabled up to 25% of the windward region to achieve adequate wind comfort conditions, making it suitable for activities such as walking and sitting. The region towards the leeward side has lower wind speeds, with a comfort class of A supporting various occupants' activities. This pattern is consistent with all configurations regardless of the buffer choice.

Surprisingly, placement of either vegetative barriers, hedges, or trees leads to amplification of wind speeds around the corners for all wind directions. This was significantly shown for the cases when the wind angle was 45° (Table 3 – (e) and (h)). For this choice of skygarden, the comfort class improves when the wind flow is perpendicular to the building's face. Larger patches of area are within classes S, E and D when at 45°, in contrast to the other cases when class A is predominant. Based on this evaluation, a modification in the skygarden design was introduced, combining the geometries of the lateral and peripheral designs. Further discussion about this design compared to both the peripheral and side skygarden is presented in the next section.

Table 4 presents the temperature distribution across the peripheral skygarden design for various barrier and wind configurations. Like the lateral skygarden design, the regions closest to the vegetative barriers achieved the greatest reduction, with up to a maximum of 2 °C drop in air temperature. There was limited variation in air temperature across the skygarden areas as most regions presented temperatures around 29.5 °C. However, compared to the lateral skygarden design, it provided slightly more reduction in temperature as some areas achieved temperatures as low

**Table 3** Contours of wind speed (mapped against the comfort criteria) taken at occupants' chest height for the peripheral skygarden

| Peripheral skygarden: wind comfort class | | | |
|---|---|---|---|
| | Vegetation type | | |
| Angle | None | Hedge | Trees |
| 0° |  (a) |  (d) |  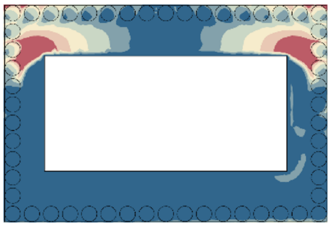 (g) |
| 45° |  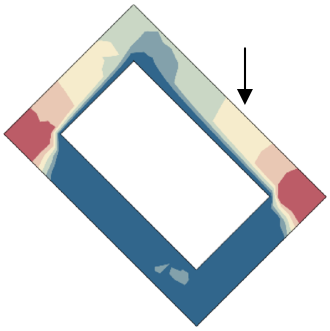 (b) |  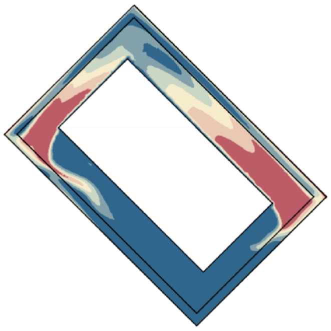 (e) |  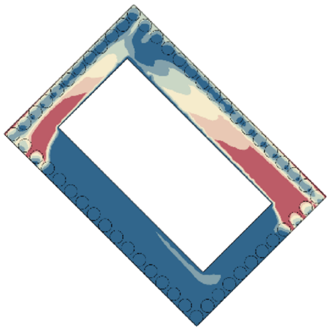 (h) |
| 90° |  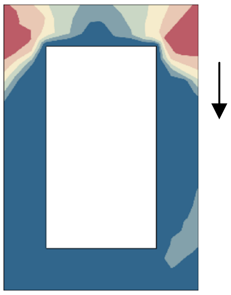 (c) |  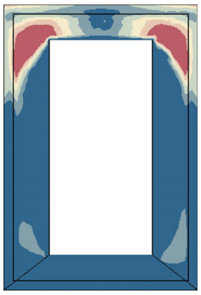 (f) |  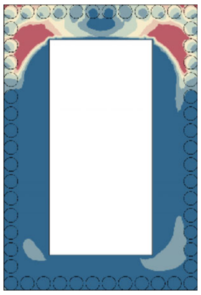 (i) |

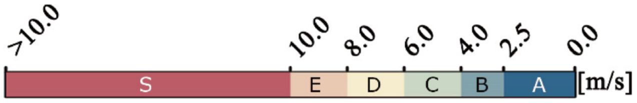

as 28.2 °C. Furthermore, this indicates that hedges have a greater potential to reduce temperature than using trees. Areas with slower wind speeds also showed more temperature drop as compared to regions with high wind speeds.

## 4.3 Combined skygarden design

The following section discusses the wind speed (Table 5) and temperature distribution (Table 6) across all cases of the combined skygarden design. In comparison to the previous two designs, a striking reduction in wind speed was observed. The area showed minimal to no wind conditions, with almost the entire region falling under the comfort class A. However, for cases when the wind direction was set at 45° to the building, wind speeds of about 4–8 m/s were observed on the windward side, viz-a-viz comfort classes of C and D (Table 5 – (b), (e) and (h)). This indicates the suitability for activities such as strolling and running, while it would not be ideal for sitting for a prolonged duration.

Furthermore, this suggests the combined skygarden design achieved the best results in terms of wind comfort as it provided the largest area with reduced wind speeds and within the comfortable range. Effectively, by breaking the peripheral skygarden design into 4 sections of lateral skygarden areas, the built mass at the corners assisted the design by obstructing airflow, enabling lower wind speeds across the skygarden regions compared to both the side and the

**Table 4** Contours of temperature taken at occupants' chest height for the peripheral skygarden

| Peripheral skygarden: temperature | | |
|---|---|---|
| | Vegetation type | |
| Angle | Hedge | Trees |
| 0° |   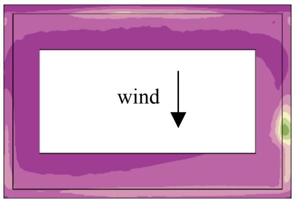 (a) |   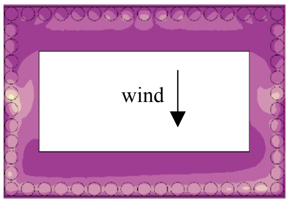 (d) |
| 45° |  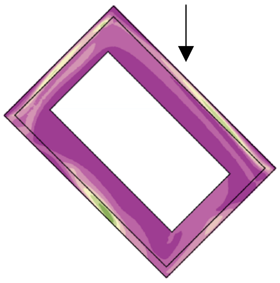 (b) |  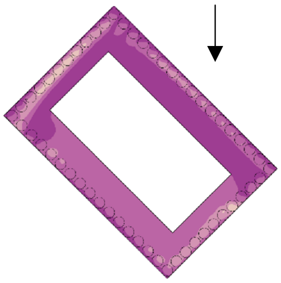 (e) |
| 90° |  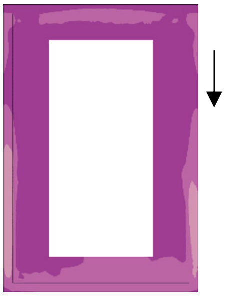 (c) |  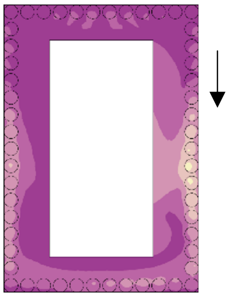 (f) |

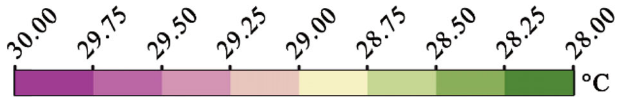

peripheral design. In contrast to previous designs, vegetative barriers in the combined skygarden design ensured reduced wind speeds for all configurations, which was not the case for some wind angles and some areas in previous designs (Table 1 – (d), (g), (e) and (h); Table 3 – (e), (h), (f) and (i)). As shown in the results for cases when the wind was at the angle of 0° and 45° to the building (Table 5 – (a), (b), (d), (e), (g), and (h)), the addition of vegetation specifically assisted in the reduction of wind flow across the windward side. Comparing images (d) with (g) and (e) with (h) (Table 5), the effect of the vegetation type can be identified by the different wind flow distributions. Overall, due to the higher influence of the skygarden design on the overall wind comfort, the addition of vegetative barriers in this design provided little advantage. However, the barriers provide relief when the wind flow is at an angle of 45°.

Whereas the vegetative barrier provided no attenuation benefits, it significantly aided in temperature reductions. It generated perceivable temperature reduction when compared to previous design alternatives. It was observed that the sides and the rear portion of the skygarden provided the most reduction in temperature, especially the sides. Variation of about 2 °C was seen in the case of the addition of trees, while about 1 °C for hedges. The windward region of the skygarden witnessed slight temperature changes in the range of 0.5 °C. This spatial configuration of the skygarden serves two important purposes. It helps achieve wind comfort by introducing a built mass as a wind deflector and allowing vegetative barriers to reduce the temperature in the vicinity.

## 4.4 Thermal comfort analysis across all skygarden designs

This section presents the thermal comfort analysis across all skygarden designs. Airspeed and temperature data were extracted from 30 points across the skygarden region of each simulation (shown in Figure 15), and the average of each case was calculated, shown in Tables 7 and 8. The choice of skygarden design, regardless of the barrier type, is the predominant factor affecting wind speeds in the region. Table 7 presents the average wind speed and quality class in the various designs. On average, all of the combined skygarden design cases fall under the quality class A, suggesting the space is suitable for leisurely activities and safe for occupants. Peripheral skygarden falls under the quality classes B and C, while lateral designs achieve higher wind speeds comparatively and belong to classes B through E. Choice of vegetative barriers plays a secondary role, with trees offering more resistance to airflow than hedges. Consequently, this provides information for architects and designers to consider when creating new highrise buildings that incorporate features of skygardens. It must be kept in mind that the implemented boundary conditions represent a typical windy day, such that the performance of the vegetation can be evaluated on the higher end.

In contrast to wind comfort, thermal variations were not significantly impacted by the choice of skygarden design, nor did the wind direction play a significant role. However, the vegetation type played a slight role. Based on the average values shown in Table 8, the air temperature was slightly lower when trees were used instead of hedges. Furthermore, the wind direction provided minimal impact on the average air temperatures. Additionally, the combined skygarden design achieved the most reduction in air temperature compared to the other two designs.

**Table 5** Contours of wind speed (mapped against the comfort criteria) taken at occupants' chest height for the combined skygarden

| Combined skygarden: wind comfort class | | | |
|---|---|---|---|
| | Vegetation type | | |
| Angle | None | Hedge | Trees |
| 0° |  (a) |  (d) |  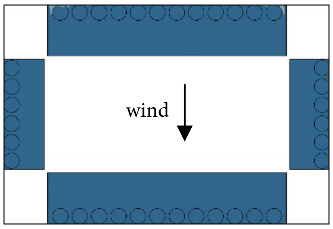 (g) |
| 45° |  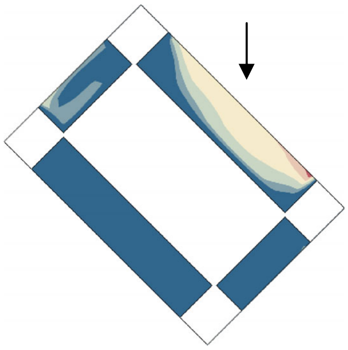 (b) |  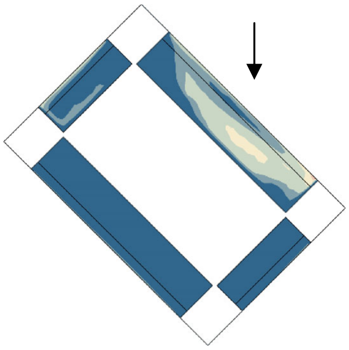 (e) |  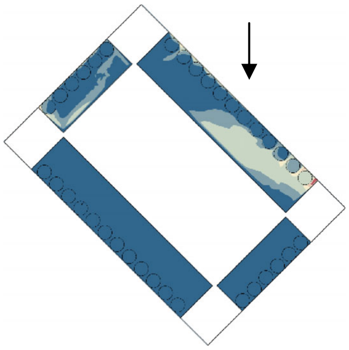 (h) |
| 90° |  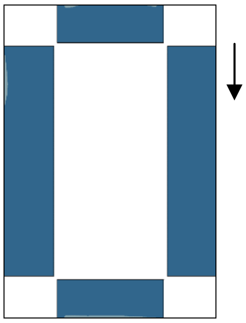 (c) |  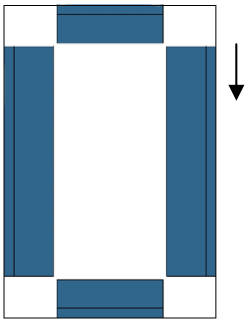 (f) |  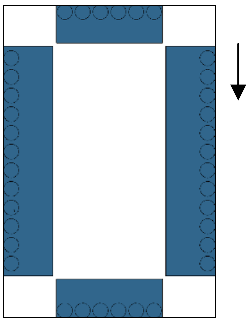 (i) |
| |  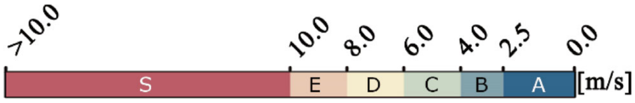 | | |

The CBE Thermal Comfort Tool based on the ASHRAE Standard 55 (ASHRAE 2020) was used to carry out a simplified thermal comfort evaluation of the skygarden configurations, based on predicted airflow speed and temperature. Since the simulation assumed a typical hot and high wind period, most of the results did not comply with the ASHRAE Standard 55 as the airspeed (shown in Table 7) was outside the range defined by the standard for occupants with no local air speed control.

The variation in the SET across the skygarden and vegetation configurations (shown in Table 9), suggests the importance of adding vegetation to the skygarden. With the wind from all simulated directions, the addition of hedges and trees could reduce SET by up to an average of 0.5 °C and 0.6 °C with the lateral skygarden design, up to 0.90 °C and 0.93 °C with the peripheral design and up to 0.80 °C and 1.1 °C with the combined design. It should be acknowledged that such predicted results were determined based on a set radiant temperature, and it did not account for factors such as shading. Therefore, future works should include analysis based on a series of different comfort factors, including the evaluation of wind comfort and the PPD values, to suggest a more complete and effective understanding of the conditions across a highrise building with different skygarden configurations.

**Table 6** Contours of temperature taken at occupants' chest height for the combined skygarden design

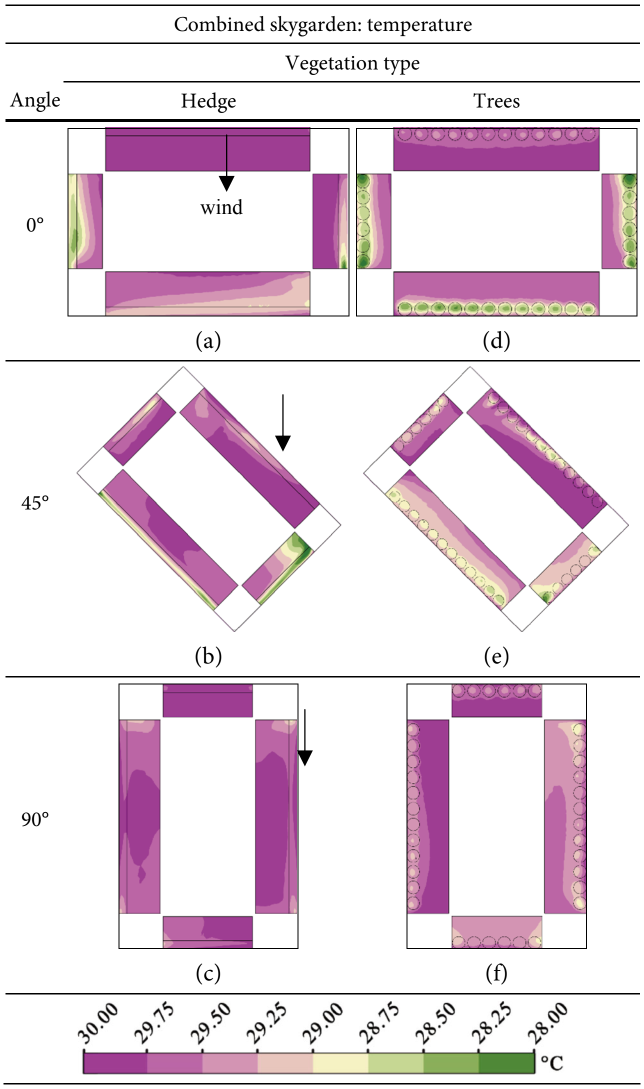

## 5 Conclusions and future works

This present study explored the aero-thermal dynamics across two skygarden configurations commonly found on highrise buildings and a proposed hybrid design. This included the lateral design in the form of open spaces on either side of a centrally located built mass. The interstitial type or the peripheral design wherein open space surrounded the built mass on all four sides, and a modified design based on a combined approach. This hybrid design consisted of a broken interstitial skygarden around the corners so that the lateral skygarden was replicated on all four sides. The numerical models were developed, and computational fluid dynamics (CFD) simulations were performed using FLUENT (ANSYS). The simulation cases included implementing vegetative elements, where hedges and trees were positioned across all edges of each skygarden area to attenuate wind speeds in typical windy conditions. Furthermore, to understand the effect of wind direction, three wind angles were simulated. This was done by orienting the building at an angle of 0°, 45° and 90° with respect to the inlet. The modified Lawson wind comfort criteria were used to assess the wind comfort conditions across the skygarden regions.

Results indicate that the spatial geometry and wind direction play an important role in determining the occupants' wind comfort level in the skygarden. In the lateral skygarden, the conditions were critical when the wind flow was perpendicular to the width of the building. The skygarden recorded dangerously high wind speeds, with values greater than 10 m/s, i.e., comfort class S. On average, this configuration generated wind speeds of 5 m/s that belong to class C, which is essentially not suitable for common activities such as sitting. However, the peripheral and the combined designs produced a conducive environment with average speeds of 3.6 m/s and 1.5 m/s, respectively. This corresponds to the comfort classes of B and C, respectively, although the vegetation played little role in generating this condition. These designs were found to be acceptable for performing common activities like sitting and strolling.

Analysis of temperature distribution across the skygardens indicated that the spatial configuration played some role in improving the thermal comfort in the skygarden. When the outdoor temperature is set to 30 °C, the configurations achieve an average SET of 25.0 °C, 24.9 °C and 25.9 °C for lateral, peripheral and combined design alternatives, respectively. On average, trees performed slightly better in reducing the

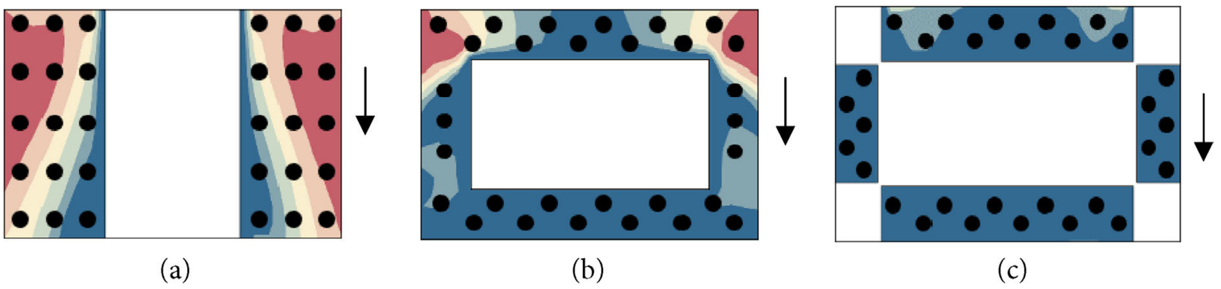

**Fig. 15** Location of the 30 selected points across the skygardens for extracting wind velocity and air temperature for the thermal comfort analysis: (a) lateral design, (b) peripheral design and (c) combined design

**Table 7** Average wind velocity across all skygarden designs and cases

| | Lateral Skygarden | | | Peripheral Skygarden | | | Combined Skygarden | | |
|---|---|---|---|---|---|---|---|---|---|
| Wind direction | None | Hedge | Trees | None | Hedge | Trees | None | Hedge | Trees |
| 0° | 8.47 | 8.00 | 7.22 | 3.08 | 3.02 | 2.63 | 1.38 | 1.51 | 1.25 |
| 45° | 4.91 | 4.24 | 4.10 | 4.24 | 5.19 | 5.17 | 2.65 | 1.83 | 1.89 |
| 90° | 3.62 | 3.23 | 3.23 | 3.08 | 3.16 | 3.23 | 1.25 | 1.25 | 1.25 |
| Average | 5.66 | 5.16 | 4.85 | 3.47 | 3.79 | 3.67 | 1.76 | 1.53 | 1.46 |

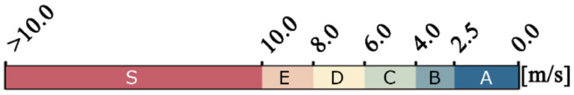

**Table 8** Average air temperature across all skygarden designs and cases

| | Lateral skygarden | | | Peripheral skygarden | | | Combined skygarden | | |
|---|---|---|---|---|---|---|---|---|---|
| Wind direction | None | Hedge | Trees | None | Hedge | Trees | None | Hedge | Trees |
| 0° | 30.00 | 29.84 | 29.83 | 30.00 | 29.34 | 29.40 | 30.00 | 29.28 | 29.06 |
| 45° | 30.00 | 29.58 | 29.38 | 30.00 | 29.33 | 29.27 | 30.00 | 29.24 | 29.00 |
| 90° | 30.00 | 29.50 | 29.51 | 30.00 | 29.45 | 29.39 | 30.00 | 29.58 | 29.27 |
| Average | 30.00 | 29.64 | 29.58 | 30.00 | 29.37 | 29.35 | 30.00 | 29.37 | 29.11 |

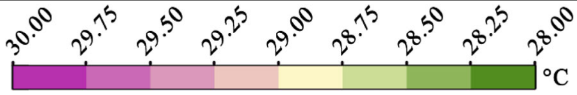

**Table 9** Predicted standard effective temperature (SET) across all skygarden designs and cases

| | Lateral skygarden | | | Peripheral skygarden | | | Combined skygarden | | |
|---|---|---|---|---|---|---|---|---|---|
| Wind direction | None | Hedge | Trees | None | Hedge | Trees | None | Hedge | Trees |
| 0° | 25.3 | 25 | 25 | 25.6 | 24.7 | 24.9 | 26.8 | 25.6 | 25.5 |
| 45° | 25.3 | 24.7 | 24.4 | 25.3 | 24.3 | 24.2 | 25.8 | 25.2 | 24.8 |
| 90° | 25.4 | 24.8 | 24.8 | 25.6 | 24.8 | 24.6 | 26.9 | 26.3 | 25.9 |
| Average | 25.3 | 24.8 | 24.7 | 25.5 | 24.6 | 24.6 | 26.5 | 25.7 | 25.4 |

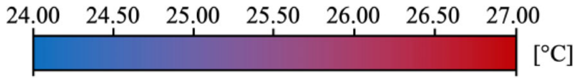

temperature of the surrounding air than hedges. Similarly, trees also performed slightly better in reducing wind speeds. Trees brought down the average wind speed and temperature by an additional 0.48 m/s and an average SET value of 0.1°C compared with the hedges.

Based on the analysis of the wind conditions in the peripheral and lateral skygardens, the building can potentially benefit from small-scale wind turbines located within the skygarden zones. The zones can be split into habitable zones and wind energy generation zones, which will benefit from accelerated winds within the skygarden. This has multiple benefits, such as making the building self-reliant to creating a conducive microenvironment. Overall, this study provides an in-depth analysis of the conditions of various skygarden designs, providing a reference case for architects and planners. The results presented here can function as a guideline to facilitate the design of a pleasant and satisfactory skygarden within a highrise building. Designers can also create zones within the space according to the need of the building and wind conditions.

The study, however, was limited in the choice of wind barriers and arrangement and assessment of structural and sustainable feasibility. Future studies should also examine other benefits of vegetative barriers, such as attenuating noise pollution, filtering air, and providing shade from solar radiation (Appendix B). A detailed model of a tree, including source terms for turbulence and moisture, will present a complete analysis of the conditions. Other skygarden designs and alternate uses of such designs remain to be explored. Detailed analysis remains to be done, including the fluctuating nature of wind flow and the impact of neighbouring structures. Finally, the thermal comfort evaluation in this study is rather simplified and mainly focused on the predicted airflow velocity and temperature, while other environmental and personal factors were fixed. Future works can also focus on conducting a comprehensive thermal comfort analysis of such spaces.

**Electronic Supplementary Material (ESM):** the Appendix is available in the online version of this article at https://doi.org/10.1007/s12273-022-0943-7.

## Acknowledgements

The support provided by the Faculty of Engineering, University of Nottingham and EPSRC is acknowledged, which includes a scholarship (EP/R513283/1) and computational facilities.

**Open Access**: This article is licensed under a Creative Commons Attribution 4.0 International License, which permits use, sharing, adaptation, distribution and reproduction in any medium or format, as long as you give appropriate credit to the original author(s) and the source, provide a link to the Creative Commons licence, and indicate if changes were made.

The images or other third party material in this article are included in the article's Creative Commons licence, unless indicated otherwise in a credit line to the material. If material is not included in the article's Creative Commons licence and your intended use is not permitted by statutory regulation or exceeds the permitted use, you will need to obtain permission directly from the copyright holder.

To view a copy of this licence, visit http://creativecommons.org/licenses/by/4.0/

## Electronic Supplementary Material

Appendix to: Numerical evaluation of the use of vegetation as a shelterbelt for enhancing the wind and thermal comfort in peripheral and lateral-type skygardens in highrise buildings

## References

[CR1] Aboelata A (2020). Vegetation in different street orientations of aspect ratio (H/W 1:1) to mitigate UHI and reduce buildings’ energy in arid climate. Building and Environment.

[CR2] Alnusairat S (2018). Approaches to skycourt design and performance in high-rise office buildings in a temperate climate.

[CR3] ANSYS (2017). ANSYS 18.2 Documentation.

[CR4] ANSYS (2022). ANSYS FLUENT 12.0/12.1 Documentation.

[CR5] ASHRAE (2010). ANSI/ASHRAE Standard 55-2010. Thermal Environmental Conditions for Human Occupancy. Atlanta, GA, USA: American Society of Heating, Refrigerating and Air-Conditioning Engineers.

[CR6] ASHRAE (2020). ASHRAE Standard 55. Thermal Environmental Conditions for Human Occupancy.

[CR7] Begeç H, Bashiri Hamidabad D (2015). Sustainable high-rise buildings and application examples. In: Proceedings of the 3rd Annual International Conference on Architecture and Civil Engineering (ACE 2015), Singapore.

[CR8] Bitog JP, Lee I-B, Hwang H-S (2011). A wind tunnel study on aerodynamic porosity and windbreak drag. Forest Science and Technology.

[CR9] Bitog JP, Lee I-B, Hwang H-S (2012). Numerical simulation study of a tree windbreak. Biosystems Engineering.

[CR10] Braun AL, Awruch AM (2009). Aerodynamic and aeroelastic analyses on the CAARC standard tall building model using numerical simulation. Computers and Structures.

[CR11] Brisbane Development (2018). Council Approves Midtown Centre Development. Available at https://brisbanedevelopment.com/council-approves-midtown-centre-development. Accessed Nov 16 2021.

[CR12] Buccolieri R, Gromke C, di Sabatino S (2009). Aerodynamic effects of trees on pollutant concentration in street canyons. Science of the Total Environment.

[CR13] CBE (2020). CBE Thermal Comfort Tool for ASHRAE-55. Available at https://comfort.cbe.berkeley.edu. Accessed Nov 16 2021.

[CR14] Chen L, Liu C, Zhang L (2017). Variation in tree species ability to capture and retain airborne fine particulate matter (PM2.5). Scientific Reports.

[CR15] Fabbri K, Ugolini A, Iacovella A (2020). The effect of vegetation in outdoor thermal comfort in archaeological area in urban context. Building and Environment.

[CR16] Ferrini F, Fini A, Mori J (2020). Role of vegetation as a mitigating factor in the urban context. Sustainability.

[CR17] Franke J (2006). Recommendations of the COST action C14 on the use of CFD in predicting pedestrian wind environment. In: Proceedings of the 4th International Symposium on Computational Wind Engineering (CWE2006), Yokohama, Japan.

[CR18] Gamero-Salinas J, Kishnani N, Monge-Barrio A (2021). Evaluation of thermal comfort and building form attributes in different semi-outdoor environments in a high-density tropical setting. Building and Environment.

[CR19] Giacomello E, Valagussa M (2015). Vertical greenery: Evaluating the high-rise vegetation of the Bosco Verticale, Milan. Chicago: Council on Tall Buildings and Urban Habitat.

[CR20] Gromke C, Ruck B (2012). Pollutant concentrations in street canyons of different aspect ratio with avenues of trees for various wind directions. Boundary-Layer Meteorology.

[CR21] Gromke C, Blocken B, Janssen W (2015). CFD analysis of transpirational cooling by vegetation: Case study for specific meteorological conditions during a heat wave in Arnhem, Netherlands. Building and Environment.

[CR22] Huang S, Li QS, Xu S (2007). Numerical evaluation of wind effects on a tall steel building by CFD. Journal of Constructional Steel Research.

[CR23] Jeanjean APR, Hinchliffe G, McMullan WA (2015). A CFD study on the effectiveness of trees to disperse road traffic emissions at a City scale. Atmospheric Environment.

[CR24] Kang G, Kim JJ, Kim DJ (2017). Development of a computational fluid dynamics model with tree drag parameterizations: Application to pedestrian wind comfort in an urban area. Building and Environment.

[CR25] Kang G, Kim JJ, Choi W (2020). Computational fluid dynamics simulation of tree effects on pedestrian wind comfort in an urban area. Sustainable Cities and Society.

[CR26] Lee RX, Jusuf SK, Wong NH (2015). The study of height variation on outdoor ventilation for Singapore’s high-rise residential housing estates. International Journal of Low-Carbon Technologies.

[CR27] Li X, Lu Q, Lu S (2016). The impacts of roadside vegetation barriers on the dispersion of gaseous traffic pollution in urban street canyons. Urban Forestry and Urban Greening.

[CR28] Liu J, Heidarinejad M, Gracik S (2015). The impact of exterior surface convective heat transfer coefficients on the building energy consumption in urban neighborhoods with different plan area densities. Energy and Buildings.

[CR29] Manickathan L, Defraeye T, Allegrini J (2018). Parametric study of the influence of environmental factors and tree properties on the transpirative cooling effect of trees. Agricultural and Forest Meteorology.

[CR30] Melbourne WH (1980). Comparison of measurements on the CAARC standard tall building model in simulated model wind flows. Journal of Wind Engineering and Industrial Aerodynamics.

[CR31] Meng F, He B, Zhu J (2018). Sensitivity analysis of wind pressure coefficients on CAARC standard tall buildings in CFD simulations. Journal of Building Engineering.

[CR32] Mohammadi M, Calautit JK (2019). Numerical investigation of the wind and thermal conditions in sky gardens in high-rise buildings. Energies.

[CR33] Mughal MO, Kubilay A, Fatichi S (2021). Detailed investigation of vegetation effects on microclimate by means of computational fluid dynamics (CFD) in a tropical urban environment. Urban Climate.

[CR34] Nazarian N, Kleissl J (2015). CFD simulation of an idealized urban environment: thermal effects of geometrical characteristics and surface materials. Urban Climate.

[CR35] Peng C, Ming T, Cheng J (2015). Modeling thermal comfort and optimizing local renewal strategies—A case study of Dazhimen Neighborhood in Wuhan City. Sustainability.

[CR36] Perini K, Magliocco A (2014). Effects of vegetation, urban density, building height, and atmospheric conditions on local temperatures and thermal comfort. Urban Forestry and Urban Greening.

[CR37] Pomeroy J (2014). The Skycourt and Skygarden: Greening the Urban Habitat.

[CR38] Rahman MA, Smith JG, Stringer P (2011). Effect of rooting conditions on the growth and cooling ability of Pyrus calleryana. Urban Forestry and Urban Greening.

[CR39] Rosenfeld M, Marom G, Bitan A (2010). Numerical simulation of the airflow across trees in a windbreak. Boundary-Layer Meteorology.

[CR40] Salim SM, Buccolieri R, Chan A (2011). Numerical simulation of atmospheric pollutant dispersion in an urban street canyon: comparison between RANS and LES. Journal of Wind Engineering and Industrial Aerodynamics.

[CR41] Salim SM, Cheah SC, Chan A (2011). Numerical simulation of dispersion in urban street canyons with avenue-like tree plantings: comparison between RANS and LES. Building and Environment.

[CR42] Santiago JL, Martín F, Cuerva A (2007). Experimental and numerical study of wind flow behind windbreaks. Atmospheric Environment.

[CR43] Sharifi A, Khavarian-Garmsir AR (2020). The COVID-19 pandemic: impacts on cities and major lessons for urban planning, design, and management. The Science of the Total Environment.

[CR44] SimScale (2019). Wind Comfort Criteria: Lawson, Davenport, and NEN 8100. Available at https://www.simscale.com/blog/2019/12/wind-comfort-criteria. Accessed 16 Nov 2021.

[CR45] Sonnenwald F, Stovin V, Guymer I, Rowiński P, Marion A (2016). Feasibility of the porous zone approach to modelling vegetation in CFD. Hydrodynamic and Mass Transport at Freshwater Aquatic Interfaces.

[CR46] Tien PW, Calautit JK (2019). Numerical analysis of the wind and thermal comfort in courtyards “skycourts” in high rise buildings. Journal of Building Engineering.

[CR47] Tominaga Y, Mochida A, Murakami S (2008). Comparison of various revised k-ε models and LES applied to flow around a high-rise building model with 1: 1: 2 shape placed within the surface boundary layer. Journal of Wind Engineering and Industrial Aerodynamics.

[CR48] Tominaga Y, Mochida A, Yoshie R (2008). AIJ guidelines for practical applications of CFD to pedestrian wind environment around buildings. Journal of Wind Engineering and Industrial Aerodynamics.

[CR49] Toparlar Y, Blocken B, Vos P (2015). CFD simulation and validation of urban microclimate: a case study for Bergpolder Zuid, Rotterdam. Building and Environment.

[CR50] Walker D (2017). Two New Biophilic Design Case Studies. Terrapin. Available at http://www.terrapinbrightgreen.com/wp-content/uploads/2015/11/Parkroyal_Case-Study.pdf. Accessed 4 Apr 2020.

[CR51] WHO (2009). WHO Guidelines for Indoor Air Quality: Dampness and Mould. World Health Organization. 23785740

[CR52] WHO (2016). Urban green spaces and health—A review of evidence.

[CR53] Wong KMG (2004). Vertical cities as a solution for land scarcity: The tallest public housing development in Singapore. URBAN DESIGN International.

[CR54] Yang A-S, Wen C-Y, Cheng C-H (2015). CFD simulations to study the cooling effects of different greening modifications. International Journal of Environmental and Ecological Engineering.

[CR55] Yang Y, Gatto E, Gao Z (2019). The “plant evaluation model” for the assessment of the impact of vegetation on outdoor microclimate in the urban environment. Building and Environment.

