# Supplementary material for: Numerical evaluation of the use of vegetation as a shelterbelt for enhancing the wind and thermal comfort in peripheral and lateral-type skygardens in highrise buildings
Source: Build Simul. 2022 Nov 1;16(2):243–61. doi: 10.1007/s12273-022-0943-7 (PMC9628446; doi:10.1007/s12273-022-0943-7)
Supplement: Supplementary file 1 — Appendix to: Numerical evaluation of the use of vegetation as a shelterbelt for enhancing the wind and thermal comfort in peripheral and lateral-type skygardens in highrise buildings [file 12273_2022_943_MOESM1_ESM.pdf]

Electronic Supplementary Material

Appendix to: Numerical evaluation of the use of vegetation as a shelterbelt for enhancing the wind and thermal comfort in peripheral and lateral-type skygardens in highrise buildings

Murtaza Mohammadi (✉), Paige Wenbin Tien, John Kaiser Calautit

Department of Architecture and Built Environment, University of Nottingham, Nottingham, UK

Supporting information to https://doi.org/10.1007/s12273-022-0943-7

Appendix A

A grid sensitivity test is performed for the baseline configuration, devoid of any skygarden and vegetation. Three meshes of increasing fineness were generated. The coarse mesh consists of 2 million cells, while the fine mesh consists of 15 million cells. Figure A1 presents a comparison of the C_p along the analysis line around the building at the height of 123 m (described in Section 2.4). On the windward face, x = [0, 1.5], the predictions are quite close; however slight deviation occurs along the rear and side face of the building, especially between the coarse and fine mesh. The results suggest that the medium mesh, with 6 million elements, is sufficient to obtain accurate results.

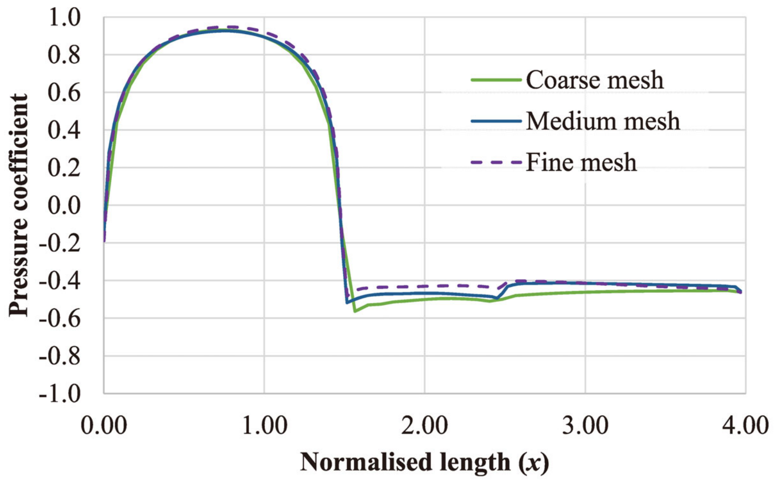

Fig. A1 Grid verification of the standard CAARC tall building design

Appendix B

The present computational model does not account for solar radiation and shading offered by vegetation. This is primarily due to the complexity of modelling the parameters within the simulation tools used in the study. However, experimental measurements have shown that vegetative strategies can provide additional thermal comfort and reduce mean radiant temperatures by blocking solar radiation. The impact can range from slight to significant benefits depending on many factors, including the tree species, outdoor conditions, and solar geometry. The impact on air temperature varies across the different studies.

E-mail: murtaza.mohammadi1@nottingham.ac.uk

Table B1 lists some studies which have attempted to quantify the thermal benefits due to solar shading mechanisms, including trees, shades and surrounding buildings. The variation between the results is large and based on context, preventing a generalised conclusion. A review by Ji et al. (2022) provides a detailed analysis of all the available thermal load calculation tools on the human skin due to radiation. They note that the various software packages provide different MRT predictions under similar scenarios. Under such circumstances, the authors decided to focus more on the wind comfort offered by vegetative measures, and a simplified thermal model is assumed. It is hoped that future work and experimental campaigns can fill this gap.

Table B1 Summary of works assessing impact of passive measures on thermal comfort (SO - semi outdoors, O - outdoors)

Reference	Location	Passive cooling/ shading strategy	Impact on T_a/MRT	Impact on thermal comfort
Acero et al. 2022	SO	Semi-outdoor sheltering, trees	T_a = ~2 °C < outdoor<br>MRT = ~33 °C < outdoor	PET ~16 °C < outdoor<br>PET ~5.9 °C < without trees
Xie et al. 2022	O	Trees and neighbouring buildings	Small difference in mean T_a,<br>T_a = 27.7 °C for less shaded pathway and T_a = 27.8 °C for better shaded pathway,<br>MRT = ~8.4 °C < less shaded pathway	TSV = 0.0–1.0 for shaded and TSV = 2.0–2.3 for direct sunlight in summer
Bouyer et al. 2007	SO	Roof canopy	—	PET up to 50 °C in direct sunlight, PET~14–22 °C in shaded area
Watanabe et al. 2014	O	Pergola (with plants) and building	Small difference in T_a,<br>T_a = 31.9 °C, 32.9 °C, and 32.9 °C for building shade, sunlight, and pergola shade	ETU reduced from 16.2 °C to 18.4 °C due to pergola (with plants) and building shades
Lee and Jim 2019	O	Green roof	Small difference in T_a,<br>T_a = 30.8 °C, and T_a = 28.7 °C for green roof under sunny weather and cloudy weather respectively	PET = 29.3 °C in sunny weather<br>PET = 27.2 °C in cloudy weather
Du et al. 2017	SO	Lift-up design	Slight difference in T_a,<br>T_a = 23.6 °C and T_a = 21.6°C under sunny and cloudy winter weather respectively,<br>MRT = 0.9 °C < sunny day	PET ~35 °C in sunny weather<br>PET ~20 °C in cloudy weather
Dissegna et al. 2021	O	Trees	MRT = 64.1°C outside tree canopy, MRT = 33.15 °C for *K. Senegalensis* with LAI = 0.1, and MRT = 25.89 °C for *K. Senegalensis* with LAI = 5.5	—
Ren et al. 2022	O	Low, medium, and high tree cover	Locations with high and medium tree cover were T_a = 5.4 °C and 2.3 °C cooler than low tree cover	Compared to low tree cover, the mean daytime cooling in PET was about 5.1 °C and 13.7 °C for the streets with medium and high tree cover
Lin and Lin 2010	O	Various species of trees with a wide range of LAI and coverage	T_a = 0.64 to 2.52 °C lower and T_s = 3.28 to 8.07 °C lower as compared to the unshaded open site	—
Huang et al. 2017	O/SO	Building shading	Small difference in T_a = 0–2 °C between shaded and open area	The neutral temperature in exposed spaces was 6.2 °C PET higher than in shaded spaces
Makaremi et al. 2012	O/SO	Pergola with plants and roofing, buildings	Moderate reduction in the T_a < 3 °C, significant reduction in MRT	The difference in PET was up to 18 °C between the two spaces
Liu et al. 2022	O	Trees, buildings	The regions with the highest T_s were non-vegetated while lowest was shaded by trees	Trees with the largest canopy diameter created cool spots that lower the PET by up to 10 °C

References

Acero JA, Ruefenacht LA, Koh EJY, et al. (2022). Measuring and comparing thermal comfort in outdoor and semi-outdoor spaces in tropical Singapore. *Urban Climate*, 42: 101122.

Bouyer J, Vinet J, Delpech P, et al. (2007). Thermal comfort assessment in semi-outdoor environments: application to comfort study in stadia. *Journal of Wind Engineering and Industrial Aerodynamics*, 95: 963–976.

Dissegna MA, Yin T, Wu H, et al. (2021). Modeling mean radiant temperature distribution in urban landscapes using DART. *Remote Sensing*, 13: 1443.

Du Y, Mak CM, Huang T, et al. (2017). Towards an integrated method to assess effects of lift-up design on outdoor thermal comfort in Hong Kong. *Building and Environment*, 125: 261–272.

Huang T, Li J, Xie Y, et al. (2017). Simultaneous environmental parameter monitoring and human subject survey regarding outdoor thermal comfort and its modelling. *Building and Environment*, 125: 502–514.

Lee LSH, Jim CY (2019). Urban woodland on intensive green roof improved outdoor thermal comfort in subtropical summer. *International Journal of Biometeorology*, 63: 895–909.

Lin BS, Lin YJ (2010). Cooling effect of shade trees with different characteristics in a subtropical urban park. *HortScience*, 45: 83–86.

Liu H, Lim JY, Thet BWH, et al. (2022). Evaluating the impact of tree morphologies and planting densities on outdoor thermal comfort in tropical residential precincts in Singapore. *Building and Environment*, 221: 109268.

Ji Y, Song J, Shen P (2022). A review of studies and modelling of solar radiation on human thermal comfort in outdoor environment. *Building and Environment*, 214: 108891.

Makaremi N, Salleh E, Jaafar MZ, et al. (2012). Thermal comfort conditions of shaded outdoor spaces in hot and humid climate of Malaysia. *Building and Environment*, 48: 7–14.

Ren Z, Zhao H, Fu Y, et al. (2022). Effects of urban street trees on human thermal comfort and physiological indices: A case study in Changchun City, China. *Journal of Forestry Research*, 33: 911–922.

Watanabe S, Nagano K, Ishii J, et al. (2014). Evaluation of outdoor thermal comfort in sunlight, building shade, and pergola shade during summer in a humid subtropical region. *Building and Environment*, 82: 556–565.

Xie Y, Wang X, Wen J, et al. (2022). Experimental study and theoretical discussion of dynamic outdoor thermal comfort in walking spaces: Effect of short-term thermal history. *Building and Environment*, 216: 109039.
